# P26 Epidemiology and risk factors associated with and clinical approach to *Staphylococcus aureus* bacteraemia at North West Anglia Foundation Trust (NWAFT)

**DOI:** 10.1093/jacamr/dlad066.030

**Published:** 2023-06-14

**Authors:** Wai Ying Katherine Wong, Dennis Mlangeni

**Affiliations:** Hinchingbrooke Hospital, North West Anglia Foundation Trust, UK; Hinchingbrooke Hospital, North West Anglia Foundation Trust, UK

## Abstract

**Background:**

*Staphylococcus aureus* bacteraemia (SAB) is associated with high mortality and morbidity. Certain patient groups are predisposed to developing SAB. Careful monitoring of these patients for persistence of bacteraemia and development of complications is important to improve prognosis. There is international consensus on (i) treating patients with at least 2 weeks of IV antibiotics, (ii) performing echocardiography to evaluate for infective endocarditis and (iii) repeating blood cultures 2–4 days after initiating empirical treatment. Adherence to trust antimicrobial guidelines is also imperative to national antimicrobial stewardship.

**Objectives:**

To characterize patients at risk of SAB and make recommendations to reduce the incidence and complications of SAB.

**Methods:**

Retrospective review of case notes of patients whose blood culture results were positive for *S. aureus* from April to September 2022 in NWAFT. 57 results from 51 patients aged 0 to 96 years were included. The four audit standards were compliance with (i) trust empirical antimicrobial guidelines, (ii) 2-week duration of IV therapy, (iii) echocardiography evaluation for endocarditis, and (iv) repeat blood cultures 48–96 hours after initiating empirical treatment. Patients who were on palliative pathway were excluded from analysis of standards two to four. Prognostic outcomes were examined in terms of hospital readmission, recurrence and mortality.

**Results:**

36 patients (70.6%) were found to harbour known risk factors, with the presence of indwelling prosthetic devices (83.3%) and type 2 diabetes (25.0%) being the most common. The most frequent sources of infection were unknown (27.5%) as well as the skin or soft tissue (17.6%). MRSA bacteraemia accounted for 10.5% of all cases. The compliances with (i) trust empirical antimicrobial guidelines, (ii) 2-week duration of IV therapy, (iii) echocardiography evaluation of endocarditis and (iv) repeat blood cultures 48–96 hours after initial blood culture were 52.8%, 75.0%, 57.8% and 66.0%, respectively. Rates of hospital readmission, recurrence and mortality were 39.5%, 11.4% and 35.3%, respectively.

**Conclusions:**

The incidence of SAB correlates with the presence of risk factors, and SAB is associated with adverse outcomes. There was partial compliance with the audited outcomes. Introduction of trust SAB guidelines to guide clinical approach and adherence to local antimicrobial prescribing guidelines are imperative to improving patient outcomes.

